# Pumpkin seed oil–supplemented diets promoted the growth productivity, antioxidative capacity, and immune response in heat-stressed growing rabbits

**DOI:** 10.1007/s11250-023-03460-3

**Published:** 2023-01-30

**Authors:** Sameh A. Abdelnour, Mohamed G. E. Metwally, Laila B. Bahgat, Mohammed A. E. Naiel

**Affiliations:** Department of Animal Production, Faculty of Agriculture, Zagazig University, Zagazig, 44519 Egypt

**Keywords:** Growing rabbits, Heat stress, Hematology, Antioxidant activity, Immunohistochemistry

## Abstract

Heat stress is the most major environmental element contributing to rabbit health problems and reduced production. It is proposed that essential oils be applied to alleviate heat stress-induced oxidative damage in rabbits. The purpose of this feeding trial was to determine the protective impact of pumpkin seed essential oil (PSO)–supplemented diets in reducing the threat of unambient temperature on growing rabbits. Five groups of 5-week-old rabbits were allocated randomly into separated galvanized wire battery. The first group was raised under normal conditions (18 ± 2 °C) and fed a control diet (control group; CNT), whereas the other four groups were exposed to high ambient temperature (38 ± 2 °C) and fed a control diet supplemented with 0 (PSO_0.0_), 0.5 (PSO_0.05_), 1.0 (PSO_1.0_), and 2.0 (PSO_2.0_) mL PSO/kg diet. Results indicated that all supplemented groups and the positive control have higher live body weight compared with the heat stress group (PSO_0.0_) at 9 weeks of age. Supplementing of PSO resulted in significant improvement in weight gain at 5–9 weeks and 9–13 weeks compared with PSO_0.0_ group. The highest feed intake was detected in PSO_0.05_ group compared with that in other groups. Both PSO_2.0_ and PSO_2.0_ groups showed the lowest feed conversion ration compared with other groups. Heat-stressed rabbits given a high dose of PSO (1 to 2 mL) had higher hemoglobin concentrations and lower white blood cell counts throughout the experiment than those given a control diet and subjected to heat stress. All hepatic and renal function parameters improved significantly in the rabbits fed a high dose of PSO as compared to the heat-stressed control group, while protein constituents were significantly higher in experimental groups fed 2 mL PSO compared with other groups. Heat-stressed rabbits administered graded amounts of PSO had the lowest plasma glucose, cortisol, thyroid, and corticosterone concentrations and were noticed to be equivalent to the control group fed unsupplemented diet and reared under normal conditions. The immunohistochemistry analysis demonstrated that rabbit groups reared under heat stress and given 2 mL PSO supplemented diets had negative caspase-3 immunoreactivity surrounding portal tract and normal structure. In conclusion, adding pumpkin seed oil up to 2 mL/kg diet for growing rabbits is indorsed to promote growth as well as antioxidant and immunological status under heat stress conditions.

## Background

The demand for livestock products is increasing, and its fast expansion in undeveloped nations has been called the “livestock revolution” (Rust and Rust 2013). The value of animal products is primarily determined by their capability to deliver more than 17% and 33% of total global kilocalorie and protein consumption, respectively (Rosegrant et al. 2009). Sudden climate change, competitive demand for land and water, and security of feed ingredient and water at a time when it is most required are all predicted to have a negative influence on livestock productivity (Hassen and Dawid 2021; Naiel et al. 2022). Meanwhile, the main four pathways described the most likely risks of climate change on farm animal health and production are diseases and stress related to high temperatures, tremendous weather regulation, the accumulation of animal production to new environments, and outbreaks of infectious diseases, particularly vector-borne diseases that are primarily reliant on environmental and climatic factors (Rojas-Downing et al. 2017). Therefore, the fundamental difficulty is to create a balance between maintaining production, domestic nutrition security, and ecological sustainability (Almeida et al. 2021).

Rabbits are extremely vulnerable to high-temperature risks due to deficiency of sufficient sweat glands which are capable of removing excess heat from their body (Abdelnour et al. 2022). Thus, the nutrient metabolism, growth, carcass criteria, and meat quality of rabbits reared under heat stress conditions are all influenced (Abdel-Wareth et al. 2018). According to the results of Zeferino et al. (2013), the neutral thermal zone for the rabbit ranges between 21 and 25 °C; however, the ideal range of ambient temperature, under controlled conditions, ranges between 16 and 22 °C, with relative humidity ranging from 60 to 70%. The increase in ambient temperature over this limit has an effect on the preservation of thermal balance, resulting in physiological alterations and abnormalities in the metabolism (Okab et al. 2008). Therefore, producers must employ effective management practices as well as develop new methods to alleviate the risks of heat stress in rabbit production.

Vitamins, essential oils, herbs, and minerals, along with other natural feed additives, have been shown to mitigate the negative impacts of heat stress on rabbits (Yousef et al. 2003; Alagawany et al. 2018; Al-Sagheer et al. 2019; Abd El-Hack et al. 2020; Hassan et al. 2021). Recently, researchers recommended that essential oils be added to rabbit diets to boost immunity, activate antioxidant status, improve digestion, stimulate growth, and have antibacterial properties (Hasan et al. 2020). Pumpkin oil is abundant in unsaturated fatty acids, particularly Omega-3 and Omega-6, which are regarded as the most vital necessary fatty acids that the monogastric cannot produce (Hajati et al. 2011). It is also used therapeutically for treating heart problems, high blood pressure reduction, and eye and skin protection (Obi et al. 2009). There has been little information about the inclusion of pumpkins or their essential oil in livestock feed and its benefits in the animal productivity and quality of meat, milk, and eggs (Achilonu et al. 2018). Therefore, the main purpose of the current trial was to evaluate the possible protective effects of pumpkin oil–supplemented diets against heat stress hazards on blood components, antioxidant activity, immunological responses, and performance of growing rabbits.

## Methods

### Gas chromatography/mass spectrometry analysis

Pumpkin seed oil (PSO) was purchased from Pure Life Company for Natural Oils, Giza, Egypt. The PSO main bioactive compounds were inspected using the gas chromatography-mass spectrometry (GC-MS) procedure as ascribed by Meng et al. (2014). The data were determined in a full scan mode (*m*/*z* 60–600). The main bioactive components were known based on their retention time and by comparing their mass spectra with those in the National Institute of Standards and Technology 2005 database, supported with tandem mass spectrometry information. The relative content of each constituent was determined by peak area normalization percentage as shown in Table 1.

**Table 1:** Retention time (RT) and peak area (%) of the different constituents found in pumpkin seed oil analyzed by gas chromatography/mass spectrometry

Compound	RT (min)	Peak area %	Library
Estragole	6.19	6.99	Mainlib
Anethole	7.82	7.74	Replib
2,4-Decadienal	8.37	2.29	Mainlib
Eugenol	9.19	3.24	Replib
1-Dodecanamine, N,N-dimethyl-	11.99	2.93	WileyRegistry8e
Phenol, 2,6-bis(1,1-dimethylethyl)-4-methyl-	12.15	1.14	WileyRegistry8e
2,2,6-trimethyl-6-(4-methyl-3-cyclohexen-1-yl) tetrahydro-2h-pyran-3-ol #	16.57	1.93	WileyRegistry8e
Anthracene	17.07	49.01	Replib
Hexadecanoic acid	20.32	2.70	WileyRegistry8e
Pyrene	22.52	14.93	Replib
Oleic acid	23.07	6.88	Replib

### Animals and experimental design

A total of one hundred and fifty healthy New Zealand white growing weaned rabbits (males, 6 weeks, and weighing 501 *±* 1.66) were included in the present research. Animals were placed in appropriate galvanized wire battery cages with standard dimensions (50 × 45 × 40 cm^3^) in a well-ventilated rabbitry. The rabbit cages were provided with manual feeders and an automatic system of nipple drinkers to offer freshwater ad libitum. Rabbits were randomly allocated into five equal groups, each of which (10 cages for each group and 3 rabbits per each cage) for eight consecutive weeks. The experiment consisted of five treatments; positive group (CNT), animals kept under thermoneutral conditions where the ambient temperature was set at 18 ± 2 °C, whereas, the other four treated groups were exposed to higher ambient temperature (38 ± 2 °C) and received a basal diet supplemented with graded levels of pumpkin seed oil (PSO) PSO_0.0,_ PSO_0.5_, PSO_1.0_, and PSO_2.0_ at 0.0, 0.5, 1, and 2 mL /kg diet, respectively. All growing rabbits were preserved under controlled conditions for 13 weeks.

**Fig. 1:**
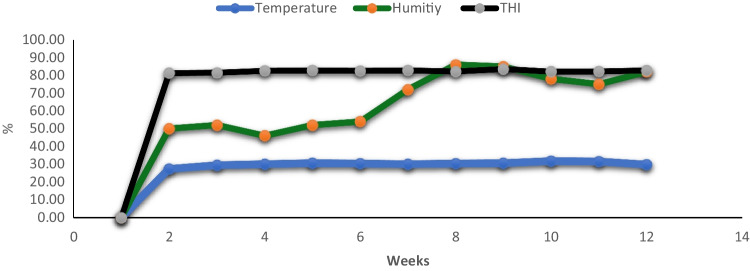
Calculated temperature-humidity index (THI) throughout the feeding trial period

The composition and nutrient content of formulated diets are presented in Table 2. The experimental diets were formulated (diameter 4 MM) to meet the nutritional requirements of growing rabbits as recommended by the De Blas and Mateos (2010). To validate the exposure of growing rabbits to heat stress condition, the average daily air temperature and relative humidity were estimated as ascribed by Müller (1989) based on estimated degrees at 09:00, 14:00 and 21:00 h. The temperature–humidity index (THI) was also calculated according to Kelly and Bond (1971) and illustrated in Fig. 1.

**Table 2:** Ingredients and composition of commercial diet for growing rabbits

Items	Basal diet
Ingredient	(%)
Soybean meal	15
Wheat bran	22
Berseem hay	30
Maize grain	10
Molasses	3
Barely grain	18
DL-methionine	0.2
Limestone	0.5
Ca (H_2_PO_4_)_2_	0.5
NaCl	0.5
Premix^1^	0.3
Total	100
Analyzed composition	(%)
Digestible energy, Cal /kg	2500
Growth energy, Cal /kg	1928.43
Crude protein	17.31
Ether extract	2.49
Crude fiber	12.34
Dry matter	88.06
Ash	9.43
Nitrogen-free extract	58.43

^1^Each 1 kg of premix (minerals and vitamins mixture) contains vit. A, 20,000 IU; vit. D_3_, 15,000 IU; vit. E, 8.33 g; vit. K, 0.33 g; vit. B_1_, 0.33; vit. B_2_, 1.0 g; vit. B_6_, 0.33 g; vit. B_5_, 8.33 g; vit. B_12_, 1.7 mg; pantothenic acid, 3.33 g; biotin, 33 mg; folic acid, 0.83 g; choline chloride, 200 g

### Growth performance

During the feeding experiment period, all growth performance metrics at 5, 9, and 13 weeks of age were assessed. The successive live body weight (BW) of growing rabbits was recorded in order to calculate the average weight gain (ABWG) for each period. Also, the consumed feed (FI) and feed conversion ratios (FCR; g feed/g gain) were also determined in each treated group (Table 3).

**Table 3:** Effects of graded level of pumpkin seed oil–supplemented diets on growth performance of NZW growing rabbits at 13 weeks of age under heat stress condition

Parameter	Experimental diets (mL/kg)					SEM	*P* value
	CNT	PSO_0.0_	PSO_0.5_	PSO_1.0_	PSO_2.0_		
Live body weight (g)							
Week 5	501.4	502.2	501.4	500.8	503.2	1.66	0.825
Week 9	1255.2^a^	1154^b^	1323.6^a^	1315.4^a^	1326^a^	12.12	0.004
Week 13	2309.2	2250.6	2316.6	2314.6	2289.4	27.41	0.456
Weight gain (g)							
Weeks 5–9	753.8^a^	651.8^b^	822.2^a^	814.6^a^	822.8^a^	16.72	0.004
Weeks 9–13	1218^b^	1126c	1298.6^a^	1293.4^a^	1305^a^	16.14	0.006
Weeks 5–13	1807.8	1807.8	1815.2	1813.8	1786.2	26.69	0.436
Feed intake (g/day)							
Weeks 5–9	37.6 ^b^	37.8^b^	45.2^a^	39.4^b^	38.2^b^	0.74	< 0.001
Weeks 9–13	48.6^b^	56.8^a^	57.0^a^	42.4^c^	40.2^c^	0.60	< 0.001
Weeks 5–13	86.2^c^	94.6^b^	102.2^a^	81.8^d^	78.4^d^	0.78	< 0.001
Feed conversion ratio (g\g BG)							
Weeks 5–9	5.81^a^	4.50^bc^	5.51^ab^	4.84^c^	4.65^c^	0.13	0.003
Weeks 9–13	3.99^c^	5.05^a^	4.39^b^	3.28^d^	3.08^d^	0.07	< 0.001
Weeks 5–13	4.77^b^	5.41^a^	5.64^a^	4.52^b^	4.39^b^	0.09	< 0.001

Different superscripts (a, b, c, and d) within each row are significantly different (*P* < 0.05). *CNT*, control, reared under optimal temperature + fed unsupplemented diet; PSO_0.0_, reared under heat stress+ fed unsupplemented diet; PSO_0.5_, reared under heat stress+ fed 0.5 mL pumpkin seed oil–supplemented diet; PSO_1.0_, reared under heat stress+ fed 1 mL pumpkin seed oil–supplemented diet; PSO_2.0_, reared under heat stress+ fed 2 mL pumpkin seed oil–supplemented diet; *SEM*, standard error of the mean; overall treatment *P* value

### Blood metabolites

At the end of the feeding trial period, blood samples were taken randomly from five animals in each group and placed into heparinized tubes. Each blood sample was divided into two equal portions. One portion was directly used for estimating the hematological variables (including RBC, red blood cells; HGB, hemoglobin; HCT, hematocrit; MCV, mean corpuscular volume; MCH, mean corpuscular hemoglobin; MCHC, the mean corpuscular hemoglobin concentration; WBC, white blood cells; lymphocytes; granulocytes; and MID) using blood hematology analyzer (HB 7021), whereas the other fraction was centrifuged for 15 min at 3000 rpm before being stored under – 20 °C for estimated stress remarks and biochemical parameters.

### Blood biochemical indices

Blood metabolites including total protein, albumin, total bilirubin, and direct and indirect bilirubin were estimated conferring to the manufacturer’s instructions by the spectrophotometric technique using commercial diagnostic kits (Bio-diagnostic Co., Giza, Egypt). The total globulin fraction was generally calculated by subtracting the albumin value from the total protein concentration. Liver enzymes (aspartate aminotransferase, AST, and alanine aminotransferase, ALT) concentrations were assessed via an automatic analyzer using a commercial kit from Bio-diagnostic Company (Giza, Egypt) as ascribed by the manufacturing procedure. Moreover, renal function indices (such as creatinine and uric acid) were measured using clinical laboratory kits that rely on spectrophotometric analysis.

### Stress remark indices

The blood glucose concentrations were measured calorimetrically using Glucose Assay Kit (Cell-Bio. Lab. Inc.) following manufacture instructions, whereas the triiodothyronine (T3) level was detected in blood using the radioimmunoassay (RIA) technique followed Chopra et al. (1996) protocol. Also, stress-related hormones such as cortisol and corticosterone were measured in blood samples applying the radioimmunoassay (RIA) methodology as ascribed by Bekhbat et al. (2018).

### Immunohistochemical examination

The hepatic specimens were fixed using graded levels of ethanol (70–100%), cleared in xylene, and finally, embedded in paraffin. Thereafter, 5-μm thickness sections were prepared and then blended with 0.01 mol/L citrate-buffered saline (pH 6.0), and quenching of endogenous peroxidase activity was performed by using H_2_O_2_ (0.3%) in phosphate-buffered saline. Then, blocking of the non-specific binding of immunological reagents was done via incubation for 60 min with normal rabbit serum 10% (*v*/*v*). Sections of the hepatocytes were incubated with caspase-3 rabbit polyclonal antibody (Abcam, Cat. Ab3235, Cambridge, UK) overnight at 4 °C. Sections were washed with PBS and incubated for 30 min with streptavidin-peroxidase conjugate (Histofine kit, Nichirei Corporation, Japan). The photomicrographs of respective tissue sections were taken using Olympus BX41 research optical microscope fitted with an Olympus DP25 digital camera, Cytology, and Histology Department, Faculty of Veterinary Medicine, Damanhur University, Egypt. The quantitative immunohistochemical detection was identified by original micrographs obtained from 5 random fields of each immune-stained section (×400) (Schneider et al. 2012).

### Statistical analysis procedure

All data collected and calculated were examined for normality and homogeneity. The post hoc Tukey’s range test using the one-way ANOVA analytic technique was applied to determine the significance between experimental treatments. The statistical significance was considered at *P* < 0.05. All the results were performed using the SPSS (V 22.0, SPSS Inc., Chicago, IL, USA). All obtained results were represented as mean ± MSE.

## Results

### Growth performance

The effect of increasing PSO levels on growth performance in stressed growing rabbits is presented in Table 3.

Dietary addition of PSO had a growth-promoting effect on all growth performance variables. Only at the period 5–9 weeks of age, PSO supplementation in heat-stressed rabbit diets significantly improved the live body weight (LBW) (*P* < 0.05). In the other words, PSO-treated groups and CNT showed higher values of LBW compared with the heat-stressed group (PSO_.0.0_) with non-significant effects at the 5 and 13 weeks of age. Rabbits received different levels of PSO exhibited higher weight gain compared with other groups (*P* < 0.05). The CTN group also presented intermediate values for weight gain, with significant improvement of weight gain. PSO_0.5_ treatment significantly improved the feed intake (FI) in all experimental periods. In addition, both PSO_1.0_ and PSO_2.0_ treatments exhibited an improvement in the FCR compared with the other groups. At 9–13 and 5–13 weeks of age, it is interesting to note that the dietary PSO_0.5_ had the higher values of FI in relation to the other groups, while the lowest values of FI were observed in PSO_1.0_ and PSO_2.0_. Heat-stressed animals fed PSO had no effects on the final BG compared with CNT and PSO_0.0_ groups. Moreover, the stressed animals received with 1 or 2 mL PSO/kg diet was more prominent compared to other levels group (Table 3).

### Blood hematological

In addition, Table 4 shows the effects of graded PSO on erythrogram and leucogram constituents. There were no significant differences in all erythrogram constituents (except HGB) in PSO-supplemented and both CNT and PSO0.0 groups. However, the leucogram constituents were decreased (*P* < 0.05) with PSO treatments compared to CNT group. The lowest values of MID and granulocytes and the highest value of lymphocytes were noticed in the PSO1.0 group. Overall, the best values of leucogram constituents were observed in the group supplemented with (1 mL/kg diet).

**Table 4:** Effects of graded levels of pumpkin seed oil–supplemented diets on blood hematological parameters of NZW growing rabbits at 13 weeks of age under heat stress condition

Parameter	Experimental diets (mL/kg)					SEM	*P* value
	CNT	PSO_0.0_	PSO_0.5_	PSO_1.0_	PSO_2.0_		
Erythrogram constituents							
RBC, 10^12^ × L	6.17	6.13	6.93	7.24	7.25	0.33	0.700
HGB, g/dL	11.69^b^	11.46^b^	12.31^ab^	13.22^a^	12.93^a^	0.26	0.016
HCT, %	38.62	47.63	41.35	35.07	37.65	3.89	0.419
MCV, fL	58.67	57.80	59.70	58.21	57.75	0.94	0.545
MCH, pg	23.87	18.84	19.91	19.60	19.08	0.72	0.124
MCHC, g/dL	30.78	32.68	30.78	33.74	33.52	0.92	0.387
Leucogram constituents							
WBC, 10^9^ × L	8.38^b^	11.49^a^	10.32^ab^	9.40^b^	8.55^b^	0.45	< 0.001
Lymphocytes, 10^9^ × L	47.08^abc^	49.30^ab^	40.07^c^	53.92^a^	46.01^bc^	1.78	< 0.001
Mid cells, 10^9^ × L	8.95^ab^	10.32^ab^	11.42^a^	7.86^b^	8.9^ab^	0.73	0.025
Granulocytes, 10^9^ × L	44.75^ab^	48.9^a^	35.01^d^	38.2^c^	42.6^bc^	1.03	< 0.001

Different superscripts (a, b, c, and d) within each row are significantly different (*P* < 0.05). *CNT*, control, reared under optimal temperature + fed unsupplemented diet; PSO_0.0_, reared under heat stress+ fed unsupplemented diet; PSO_0.5_, reared under heat stress+ fed 0.5 mL pumpkin seed oil–supplemented diet; PSO_1.0_, reared under heat stress+ fed 1 mL pumpkin seed oil–supplemented diet; PSO_2.0_, reared under heat stress + fed 2 mL pumpkin seed oil–supplemented diet; *RBC*, red blood cells; *HGB*, hemoglobin; *HCT*, hematocrit; *MCV*, mean corpuscular volume; *MCH*, mean corpuscular hemoglobin; *MCHC*, the mean corpuscular hemoglobin concentration; *WBC*, white blood cell; *SEM*, standard error of the mean; overall treatment *P* value

### Changes in the blood proteins, liver, and renal functions

Rabbits who received PSO at any level had significant differences in the protein fractions and the liver and kidney functions (Table 5). Dietary addition of PSO increased (*P* < 0.05) plasma TP, ALB, and GLOB levels compared to both CNT and PSO0.0 groups; the marked increase was observed in PSO_2.0_ group. However, there was no significant change in GLOB between PSO_1.0_ and PSO_2.0_ groups (Table 5). For ALB/GLOB ratio, CNT group exhibited the higher values followed by PSO0.0; however, there was no significant change due to PSO dietary inclusion. Significant higher (*P* < 0.05) levels of TB, ALT, AST, and creatinine were recorded in CNT; however, there were no significant differences observed among PSO0.0 and other PSO groups. Both PSO1.0 and PSO2.0 groups showed a significant increase in IDB levels compared to the other groups. Dietary inclusion of PSO decreased significantly (*P* < 0.001) uric acid compared with the CNT group; however, the lowest values were noticed in PSO0.0 group (Table 5).

**Table 5:** Effects of graded levels of pumpkin seed oil–supplemented diets on serum biochemical indices of NZW growing rabbits at 13 weeks of age under heat stress condition

Parameter	Experimental diets (mL /kg)					SEM	*P* value
	CNT	PSO_0.0_	PSO_0.5_	PSO_1.0_	PSO_2.0_		
Protein constituents							
TP, g dL^-1^	4.67^e^	5.52^d^	6.44^c^	6.71^b^	7.21^a^	0.19	< 0.001
ALB, g dL^-1^	3.39^d^	3.76^c^	4.13^ab^	4.06^b^	4.26^a^	0.20	< 0.001
GLOB, g dL^-1^	1.28^d^	1.77^c^	2.31^b^	2.65^a^	2.95^a^	0.16	0.027
ALB\GLOB ratio	2.64^a^	2.12^b^	1.79^bc^	1.53^c^	1.44^c^	0.23	0.015
Liver and kidney function indices							
TB, mg dL^-1^	1.19^a^	0.58^bc^	0.58^bc^	0.52^c^	0.64^b^	0.26	0.011
DB, mg dL^-1^	0.20^a^	0.12^c^	0.15^b^	0.14^bc^	0.16^b^	0.92	< 0.001
IDB, mg dL^-1^	0.39^b^	0.46^b^	0.43^b^	0.94^a^	0.72^a^	0.28	0.003
ALT, IU	70.55^a^	34.70^d^	49.32^b^	34.46^d^	43.93^c^	0.83	< 0.001
AST, IU	231.4^a^	103.8^b^	111.2^b^	114.4^b^	117.9^b^	3.05	< 0.001
Creatinine, mg dL^-1^	1.43^a^	1.10^b^	1.16^b^	1.12^b^	1.10^b^	0.82	< 0.001
Uric acid, mg dL^-1^	5.10^a^	2.72^d^	3.93^c^	4.36^b^	3.84^b^	0.36	< 0.001

Different superscripts (a, b, c, d, and e) within each row are significantly different (*P* < 0.05). *CNT*, control, reared under optimal temperature + fed unsupplemented diet; PSO_0.0_, reared under heat stress+ fed unsupplemented diet; PSO_0.5_, reared under heat stress+ fed 0.5 mL pumpkin seed oil–supplemented diet; PSO_1.0_, reared under heat stress+ fed 1 mL pumpkin seed oil–supplemented diet; PSO_2.0_, reared under heat stress + fed 2 mL pumpkin seed oil–supplemented diet; *TP*, total protein; *ALB*, albumin; *GLOB*, globulin; *TB*, total bilirubin ;*DB*, direct bilirubin; *IDB*, in direct bilirubin; *ALT*, alanine aminotransferase; *AST*, aspartate aminotransferase; *SEM*, standard error of the mean; overall treatment *P* value

### Stress biomarkers

Glucose was improved significantly by PSO (0.5 or 1 mL/kg diet) in stressed rabbits compared with the PSO0.0 and PSO2.0 groups, while the CNT group showed the highest values (*P* < 0.001) of glucose (Table 6). As shown in Table 6, PSO administration significantly reduced the plasma T3 compared with the PSO0.0 group; however, the CNT recorded the lowest values of T3. Dietary addition of PSO improved significantly stress hormones such cortisol and corticosterone levels in stressed rabbits compared to the PSO0.0 group; the marked decrease was observed in CNT group. Compared with the CNT group, there were no significant differences in cortisol (*P* < 0.001) and corticosterone (*P* < 0.05) levels regarding PSO0.5 and PSO2.0 groups, respectively. Overall, PSO administration significantly improved the stress biomarkers especially hormonal alterations compared to the CNT group.

**Table 6:** Effects of graded level of pumpkin seed oil–supplemented diets on serum stress remarks of NZW growing rabbits at 13 weeks of age under heat stress condition

Parameter	Experimental diets (mL/ kg)					SEM	*P* value
	CNT	PSO_0.0_	PSO_0.5_	PSO_1.0_	PSO_2.0_		
Glucose, ng dL^-1^	111.4^a^	33.56^d^	97.24^b^	72.89^c^	38.34^d^	2.42	< 0.001
Thyroid (T_3_), μg dL^-1^	0.32^d^	1.57^a^	0.90^b^	0.70^bc^	0.51^d^	0.19	0.021
Cortisol, ng mL^-1^	5.11^d^	17.40^a^	5.29^d^	8.65^c^	13.76^b^	0.26	< 0.001
Corticosterone, pg mL^-1^	0.32^d^	2.97^a^	1.68^b^	0.72^c^	0.42^d^	0.17	0.032

Different superscripts (a, b, c, and d) within each row are significantly different (*P* < 0.05). *CNT*, control, reared under optimal temperature + fed unsupplemented diet; PSO_0.0_, reared under heat stress+ fed unsupplemented diet; PSO_0.5_, reared under heat stress+ fed 0.5 mL pumpkin seed oil–supplemented diet; PSO_1.0_, reared under heat stress+ fed 1 mL pumpkin seed oil–supplemented diet; PSO_2.0_, reared under heat stress + fed 2 mL pumpkin seed oil–supplemented diet; *SEM*, standard error of the mean; overall treatment *P* value

### Immunohistochemical findings

**Fig. 2:**
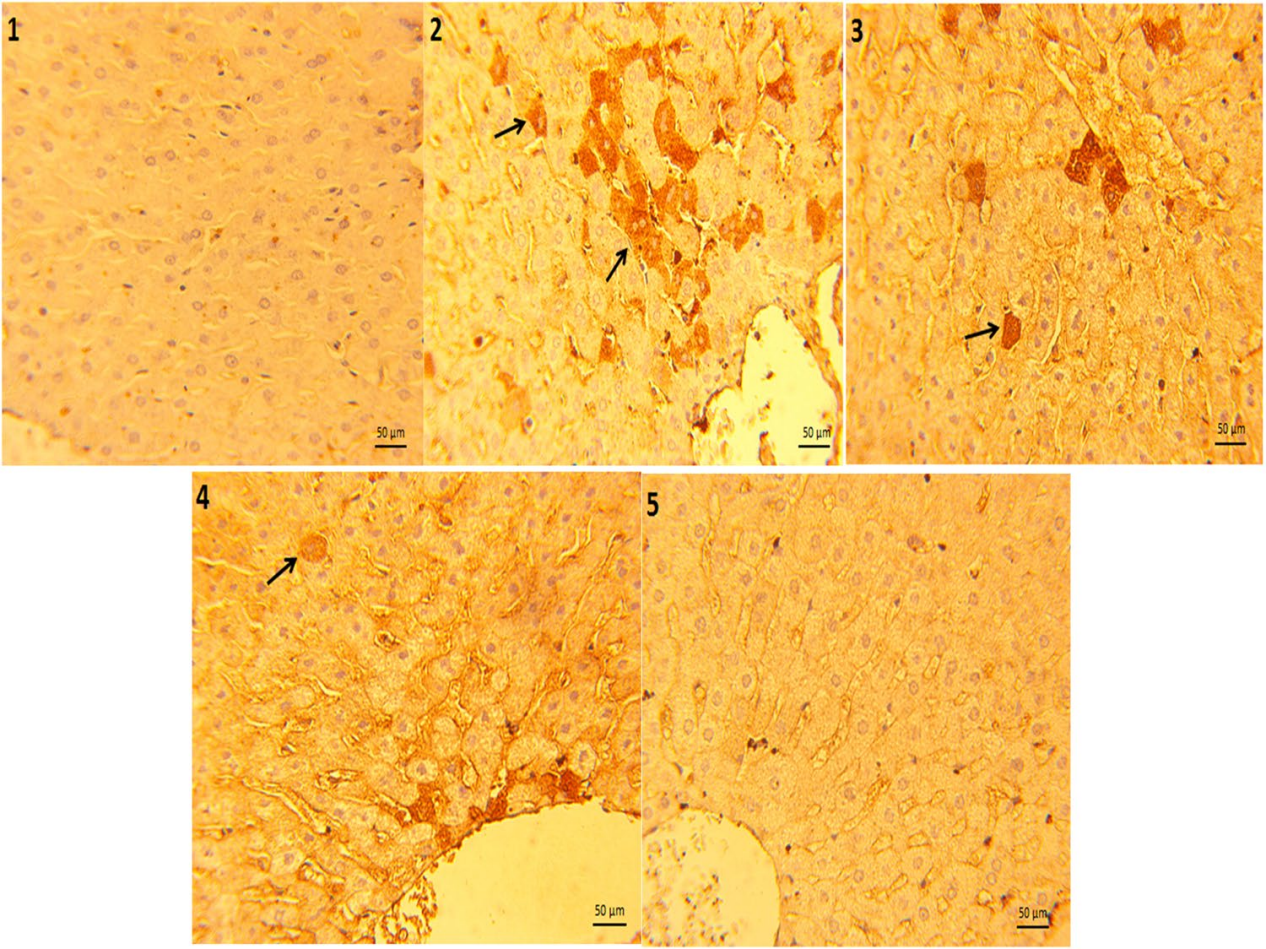
Growing rabbit liver-stained sections (50 μm) of immunoreactivity for caspase-3 showing (1) negative immune reaction for caspase-3 in the hepatocytes surrounding portal tract (PT) with no observing of any immuno-stained cells (normal structure). (2) Rabbit groups reared under heat stress and fed unsupplemented diet illustrating intense immunoreactivity for caspase-3 in the cytoplasmic remnants of the vacuolated hepatocytes surrounding PT (arrows). (3) Rabbit groups reared under heat stress and fed 0.5 mL PSO enriched diets showing mild positive numbers of caspase-3 immunoreactivity within hepatocytes (arrows) with hepatocytes exhibiting mild caspase-3 cytoplasmic immunoreactivity with vesicular nuclei. (4) Rabbit groups reared under heat stress and fed 1 mL PSO enriched diets showing a weak cytoplasmic positive caspase-3 immunoreactivity around PT (arrows). (5) Rabbit groups reared under heat stress and fed 2 mL PSO enriched diets observing negative caspase-3 immunoreactivity around PT and showing normal structure (caspase-3 × 400)

Figure 2 shows the immunohistochemistry for caspase-3 in growing rabbit liver-stained sections. The control group fed unsupplemented diet and raised under normal conditions exhibits immune reaction for caspase-3 in the hepatocytes surrounding portal tract (PT) with no observing of any immuno-stained cells (normal structure), while rabbit groups reared under heat stress and fed unsupplemented diet illustrating intense immunoreactivity for caspase-3 in the cytoplasmic remnants of the vacuolated hepatocytes surrounding PT, whereas the groups reared under heat stress and fed 0.5 mL PSO enriched diets demonstrating mild positive numbers of caspase-3 immunoreactivity within hepatocytes and hepatocytes exhibiting mild caspase-3 cytoplasmic immunoreactivity with vesicular nuclei. Moreover, rabbit groups reared under heat stress conditions and fed diets supplemented with PSO at level 1 mL/kg representing a weak cytoplasmic positive caspase-3 immunoreactivity around PT. Finally, the experimental group was grown under high-ambient temperature and fed diets containing 2 mL PSO/kg indicating negative caspase-3 immunoreactivity around PT and showing normal histological structure.

## Discussion

Heat stress produces various severe alterations in physiological functions, which are responsible for the deficiencies in rabbit production and reproduction (Mutwedu et al. 2021). The feeding trial results revealed that higher temperatures (38 °C) had an influence on several growth metrics, such as final body weight and body weight gain, while feed conversion ratio (FCR) increased in rabbits exposed to heat stress compared to the control group reared under normal conditions (18 °C), whereas the rabbits fed different inclusion levels of PSO recorded the higher with gain at 9 weeks of age. At 9 or 13 weeks of age, both levels of PSO (1 or 2) presented significant decreased in feed intake and improvement the feed conversion ratio in the rabbits exposed to heat stress. These findings were consistent with those of Abdel-Wareth et al. (2018), who discovered that thyme oil could be applied to rabbit rations at a rate of up to 100 mg/kg blended with 1.50 g/kg olive oil for enhancing rabbit performance under heat stress conditions. The enhancement in rabbit growth performance and feed efficiency under heat stress conditions might be attributed to active compounds present in PSO, mainly eugenol (Jang et al. 2007; Hong et al. 2012). Eugenol, commonly known as clove oil, is an aromatic oil that is extensively applied as a dietary flavor and might be used orally to treat digestive and respiratory disorders (Shakeel et al. 2021). Thus, treatment of rabbits with eugenol-based oil may improve antioxidant enzyme efficiency against free radicals, intestinal microbial population, and digestive enzyme activity, then indirectly enhance performance and general health status (Mu’nisa et al. 2015).

According to our findings, rabbits subjected to heat stress conditions had lower levels of Hb, higher levels of WBCs, and a lower proportion of WBCs when compared to rabbits reared under normal conditions. These results are consistent with those of previous studies that discovered that exposing growing rabbits to high ambient temperatures impaired most hematological parameters (Ondruska et al. 2011; Khalil et al. 2015; Abdelnour et al. 2020; Abdelnour et al. 2022). Also, numerous studies have shown that stress indicators such as glucose and stress hormones like thyroid, cortisol, and corticosterone caused significant changes in the absolute numbers and relative constituents of WBCs in the blood (Mommsen et al. 1999; Johnstone et al. 2012). Essential oils are often claimed to play some defensive roles such as thermoregulation, light reflectance, decreased water loss, and having antimicrobial activity as well as promoting general health status (Sharifi-Rad et al. 2017). Our feeding trial results indicate that hemoglobin, WBC count, and WBC components improved significantly in rabbits exposed to high ambient temperature and administered graded amounts of PSO in a dose-dependent manner. These findings might be attributed to the PSO components’ beneficial impact on lowering RBC lysis under stressful situations, resulting in higher hemoglobin levels in the blood (Wahab et al. 2010). Physiologically, there is a negative correlation between the levels of thyroid hormone and WBC count known as neutrophils in blood (Parma et al. 1997). Low neutrophil levels often raise the risk of severe infections (Lantin et al. 2011). Our results exhibited that PSO significantly lowered thyroid levels in the blood to near-normal levels, demonstrating PSO’s effectiveness as an anti-heat stress agent through regulating the thyroid-WBC pathway.

According to the facts provided above, elevated cortisol levels caused by high ambient temperature may encourage the thyroid to convert into an inactive form, increasing reverse T_3_ levels rather than converting free T_4_ to free T_3_, which is essential for glucose control because it impacts the number of insulin receptors available and their receptivity to insulin (Orlov et al. 2015). Our feeding experiment findings showed significantly decreased blood glucose, cortisol, and corticosterone concentrations in rabbits subjected to heat stress and administered PSO-enriched meals in a dose-dependent manner. These findings were found to be consistent with Daader et al. (2018) findings that enriched growing rabbit diets with lemongrass essential oil (LGEO) alleviated heat stress risks by modulating several physiological features. Furthermore, the inclusion of essential oregano oil in white New-Zealand rabbit diets may increase rabbit well-being while reducing stress-related threats (Ayoub et al. 2021).

Acute stress, whether prolonged or temporary, may cause measurable changes in blood total protein and its components (Coeurdacier et al. 2011). Further, the elevation in blood urea and creatinine levels associated with renal dysfunction might be due to higher catabolism of protein caused by a significant stimulation of the generation of the enzyme arginase under higher temperature, which interacts with urea synthesis (Yanardag et al. 2007). While the activity of liver enzymes is reliant on the amino acid groups alanine and glutamine adsorbed by the hepatocytes, it also reflects a dysfunction in liver metabolism linked with gluconeogenesis (El-Maghawry et al. 2000). Our biochemical blood results demonstrated that high-PSO-enriched rabbit diets remarkably improved all liver and renal enzyme activity under hot climate conditions. Furthermore, higher levels of PSO incorporation in rabbit diets increased total protein, albumin, and globulin concentrations under high ambient temperatures. The present findings are consistent with Abdelnour et al. (2022) fundings that thyme-based diets have a vital role in enhancing rabbit behavior and performance as well as moderating the harmful effects of heat stress on the liver and renal function enzymes. Also, Elazab et al. (2022) reported that dietary rosemary and ginger essential oils were effective in promoting the activity of the liver and renal function enzymes in growing rabbits, which agreed with our findings. In short-term studies, pumpkin seed oil has been shown to reduce the accumulation of fats within liver tissue and blood arteries or veins. In brief, the possible explanation is that PSO may have hepatoprotective benefits by scavenging free radicals, quenching their harmful effects, and treating liver damage (Rouag et al. 2020). Furthermore, pumpkin seeds are rich in potassium and phosphorus, and pumpkin puree is high in potassium, both of which might be employed to activate renal function and treat kidney diseases (K Ramadan et al. 2016).

Caspase-3 is a cysteine–aspartic acid protease that kills cells by cleaving cellular targets. Also, caspase-3 is helpful in detecting cell death and is required for apoptotic chromatin condensation and DNA fragmentation in all cell types (Liu et al. 2021). Herein, there is evidence that heat stress could induce apoptotic death in cells through the mitochondrial pathway (Gu et al. 2014). Our immunohistochemical investigation of liver-stained sections indicated that the rabbits given unsupplemented diets had high positivity for caspase-3 in the cytoplasmic remnants of the vacuolated hepatocytes surrounding the portal tract under hot ambient temperature, while rabbits administered PSO-rich diets were able to reduce heat stress hazards on liver tissue while also exhibiting negative caspase-3 immunoreactivity around the portal tract and no histopathological signs. To our knowledge, no publication reports the effects of PSO on immunoreactivity for caspase-3 of growing rabbits under hot climate conditions. Overall, Lopez-Novoa et al. (2011) reported that pumpkin supplementation reduced apoptosis via promoting *BCL-2* and reducing *BAX* gene expression levels. The regulating routes between two essential members of the *BCL-2* family (*BCL-2* and *BAX*) allow the cell to prevent cytochrome c from the liberation process, resulting in the deactivation of caspases 9 and 3 and eventually protecting the living cell from apoptosis (Wu et al. 2017).

## Conclusion

The results of this research showed that exposing growing rabbits to 38 °C for 13 weeks affected their growth performance, induced blood hematological and biochemical changes, and caused certain inflammations and stress abnormalities. Furthermore, heat stress’s negative effects might be mitigated by utilizing a 2 mL PSO/kg diet as a safe feed addition, antioxidant, and anti-inflammation agent. However, further research is required to identify PSO-protecting mechanisms that alleviate the impact of heat stress on oxidative stress and physiological damage in rabbits.

## Data Availability

The datasets presented and/or applied during the current study are available from the corresponding author on reasonable request.
